# Performance evaluation of an indirect immunofluorescence kit for the serological diagnosis of dengue

**DOI:** 10.31744/einstein_journal/2020AO5078

**Published:** 2019-12-10

**Authors:** Karina Emy Arai, Carolina Rodrigues Dal Bo, Ana Paula Marques Aguirra da Silva, Silvia Sanches Rodrigues, Cristóvão Luis Pitangueira Mangueira

**Affiliations:** 1 Faculdade Israelita de Ciências da Saúde Albert Einstein, Hospital Israelita Albert Einstein, São Paulo, SP, Brazil; 2 Hospital Israelita Albert Einstein, São Paulo, SP, Brazil

**Keywords:** Arbovirus infections, Dengue, Enzyme-linked immunosorbent assay, Serologic tests, Fluorescent antibody technique, indirect

## Abstract

**Objective:**

To evaluate the performance of indirect immunofluorescence for serological diagnosis of dengue virus in a population with high prevalence of arboviruses.

**Methods:**

Two-hundred serum samples from patients with clinical suspicion of dengue fever were tested by immunoenzymatic and indirect immunofluorescence assay BIOCHIP® mosaic. Specificity, sensitivity and Kappa coefficient were calculated. Discordant samples were tested by polymerase chain reaction for confirmation.

**Results:**

Of the 200 samples, 20% were positive and 80% negative for anti-dengue virus IgM antibodies in the immunoenzymatic test. Of the 40 positives, 25% were negative in indirect immunofluorescence. Of these ten discordant results, only 20% were also negative in the polymerase chain reaction (PCR). Of the 160 negatives in the immunoenzymatic test, 5% were positive in indirect immunofluorescence. Of these nine discordant results, 33% were positive in the PCR. The Kappa coefficient was 0.7 (0.572-0.829). Sensitivity and specificity of indirect immunofluorescence were respectively 75% and 94%. For anti-dengue virus IgG antibodies, of the 200 samples, 15.5% were positive and 84.5% were negative in the immunoenzymatic test. Of the 31 positives, 12.9% were negative in indirect immunofluorescence. Of these four discordant results, 25% were negative in the PCR. Of the 169 negatives, 8% were positive in indirect immunofluorescence. Of these 14 discordant results, 64% were also positive in the PCR. The Kappa coefficient was 0.695 (0.563-0.83). Sensitivity and specificity of indirect immunofluorescence were 87.1% and 91.7%, respectively.

**Conclusion:**

For diagnosis of acute infection, the immunoenzymatic test is enough, and the use of additional methods is not warranted. Replacing the immunoenzymatic test by indirect immunofluorescence would compromise the sensitivity for IgM. However, indirect immunofluorescence can distinguish three arboviruses simultaneously, an advantage during concomitant epidemics.

## INTRODUCTION

Dengue fever, an arbovirus infection predominantly transmitted by vectors of the *Aedes aegypti* species, is a serious public health issue in Brazil, with seasonal epidemics virtually across the entire national territory.^(^^1^^)^ The country recorded 572,308 probable cases in 2014; 1,621,797 in 2015; 1,483,623 in 2016; 251,711 in 2017 and 265,934 in 2018.^(^^2^^–^^4^^)^ All four serotypes of the dengue virus are present in Brazil.

Laboratory screening for the dengue virus mostly involves techniques of viral isolation, identification of dengue virus (DENV)-specific antibodies using serologic tests, direct identification of viral RNA, and detection of the NS1 antigen.^(^^5^^–^^7^^)^

The serologic diagnosis of acute infection is based on detection of DENV-specific immunoglobulin M (IgM), detectable in 93% of cases, 6 to 10 days after the onset of fever.^(^^8^^)^ Dengue virus-specific immunoglobulin G (IgG) can be detected in current infections if, at the time the test is performed, seroconversion has already taken place, and is otherwise useful to check for past infections. The IgG avidity test helps differentiate between primary and secondary infections by the dengue virus.^(^^6^^)^

The Enzyme-Linked Immunosorbent Assay (ELISA) is currently the most commonly used serologic test in clinical laboratories. It is a simple, quick test requiring limited high-tech equipment.^(^^9^^–^^11^^)^ During an epidemic, the ELISA assay can quickly determine the extent of transmission. In dengue-endemic areas, this test can be used to screen a large number of serum samples at low costs.^(^^9^^)^

Indirect immunofluorescence is another serologic method to identify dengue virus-specific IgM and IgG antibodies, however few studies^(^^12^^,^^13^^)^ have investigated the use of this test. Most serologic tests available in clinical laboratories in Brazil were developed abroad, and their validation studies were frequently conducted in populations in which the disease is not highly prevalent and which have not experienced concomitant outbreaks of other arboviruses, such as Zika and Chikungunya, and this could lead to false-positive results for dengue virus due to cross-reactive antibodies, hindering diagnosis.^(^^14^^)^

During the dengue fever epidemics in 2016, a new indirect immunofluorescence (IIF) test, the BIOCHIP^®^ mosaic developed in Germany and promising to serologically detect the dengue, Zika and Chikungunya viruses, was released in Brazil to compete with ELISA assays, which had been used as routine for a longer time.

## OBJECTIVE

To evaluate the diagnostic performance of an indirect immunofluorescence assay for serologic diagnosis of the dengue virus in a population with high prevalence of arboviruses, in comparison with the ELISA serologic test.

## METHODS

We used 200 serum samples from routine testing at the Clinical Laboratory of *Hospital Israelita Albert Einstein* (HIAE), collected in 2014 and sent to the laboratory due to clinical suspicion of infection by the dengue virus. Samples were characterized at the time as negative or positive for dengue virus using ELISA (Foccus, USA). All samples were tested with the BIOCHIP^®^ mosaic IIF technique (Euroimmun, Germany).

The appropriate statistical tools (EP Evaluator software) were used to calculate pre-test probabilities: sensitivity (diagnostic test's ability to detect true positive) and specificity (diagnostic test's ability to detect true negative). We also calculated post-test probabilities: positive predictive value (rate of patients with positive tests who effectively have the disease according to the gold standard test), negative predictive value (rate of patients with negative tests who effectively do not have the disease according to the gold standard test). Finally, we calculated accuracy, which is the probability of the test providing correct results, and the Kappa coefficient, a measure of the level of agreement between two methods, adjusted by the odds, *i.e.,* it informs the non-random chance, ranging from −1 to 1, where 0.00 is no agreement, 0.00-0.20 is poor agreement, 0.21-0.40 fair agreement, 0.41-0.60 moderate agreement, 0.61-0.80 good agreement, 0.81-0.99 very good agreement, and 1 is perfect agreement.^(^^15^^,^^16^^)^

In samples for which the two methods were discordant, we used polymerase chain reaction (PCR) as a confirmatory diagnostic method. Polymerase chain reaction is a molecular assay which quantitatively detects DENV RNA, and is considered the gold standard, since it can effectively prove that the virus is present in the body. However, it has limitations, including its high cost, which prevents it from being routinely used for screening in laboratories.^(^^5^^)^

This study was approved by the Institutional Review Board of *Hospital Israelita Albert Einstein*, CAAE: 83521718.5.0000.0071, opinion nº 2.909.625.

## RESULTS

Of the 200 samples studied, 40 (20%) were classified as positive and 160 (80%) as negative for anti-DENV IgM antibodies, in the reference ELISA test.

Of the 40 positive samples, 10 (25%) were negative in the IIF test; of these 10 discordant samples, only 2 (20%) were also negative in the PCR. However, of the 160 negative samples in the reference ELISA, 9 (5%) were positive in the IIF test; of these 9 discordant samples, 3 (33%) were positive for DENV in the PCR (Table 1).

**Table 1:** Detection of anti-dengue virus IgM antibodies in the ELISA and indirect immunofluorescence tests

IgM indirect immunofluorescence	IgM ELISA		Total
	Positive	Negative	
Positive	30	9	39
Negative	10	151	161
Total	40	160	200

When the Kappa test was used on results from both assays, an agreement level of 0.7 (0.572-0.829) was found. Sensitivity and specificity of indirect immunofluorescence was 75% and 94%, respectively. The positive predictive value of IIF was 76.9%, the negative predictive value was 93.7%, and the accuracy, 90.5%.

For anti-DENV IgG antibodies, of the 200 serum samples, 31 (15.5%) were classified as positive and 169 (84.5%) as negative, in the ELISA test. Of the 31 positive samples, 4 (12.9%) were negative in the IIF test; of these 4 discordant samples, only 1 (25%) had undetectable DENV in the PCR. However, of the 169 negative samples, 14 (8%) were positive in the IIF test; of these 14 discordant samples, 9 (64%) were also positive in the PCR (Table 2).

**Table 2:** Detection of anti-dengue virus IgG antibodies. Comparison between ELISA and indirect immunofluorescence results

IgG indirect immunofluorescence	IgG ELISA		Total
	Reagent	Non reagent	
Reagent	27	14	41
Non reagent	4	155	159
Total	31	169	200

When the Kappa test was used on results from both assays, an agreement level of 0.695 (0.563-0.83) was found. The sensitivity and specificity of indirect immunofluorescence was 87.1% and 91.7%, respectively. The positive predictive value of IIF was 65.8%, the negative predictive value was 97.4%, and accuracy, 91%.

## DISCUSSION

The level of agreement, as verified by the Kappa coefficient, between the new IIF test and the test used as reference ELISA was acceptable, attesting to the good performance of the new test.

However, PCR, which was the method used to confirm the presence of viral antigens in samples for which the two methods were discordant, showed higher agreement with ELISA in most cases, except those with negative ELISA and positive IIF for anti-DENV IgG antibodies (false-positive IgG), where the PCR was 64% concordant with indirect immunofluorescence.

## CONCLUSION

Indirect immunofluorescence has acceptable performance, however, for clinically relevant situations, when diagnosing acute infection (detection of IgM antibodies), ELISA alone is sufficient for serologic diagnosis, and the use of an additional method is not warranted. Replacing ELISA with indirect immunofluorescence, in turn, could compromise diagnostic sensitivity, increasing the number of false-negative samples for IgM.
