# Supplementary material for: Performance evaluation of an indirect immunofluorescence kit for the serological diagnosis of dengue
Source: Einstein (Sao Paulo). 2019 Dec 10;18:eAO5078. doi: 10.31744/einstein_journal/2020AO5078 (PMC6910063; doi:10.31744/einstein_journal/2020AO5078)
Supplement: Translation

# Translation: Avaliação do desempenho de *kit* de imunofluorescência indireta para o diagnóstico sorológico de dengue

**Authors:** Karina Emy Arai, Carolina Rodrigues Dal Bo, Ana Paula Marques Aguirra da Silva, Silvia Sanches Rodrigues, Cristóvão Luis Pitangueira Mangueira

**Affiliations:** 1 Faculdade Israelita de Ciências da Saúde Albert Einstein, Hospital Israelita Albert Einstein, São Paulo, SP, Brasil; 2 Hospital Israelita Albert Einstein, São Paulo, SP, Brasil

**Keywords:** Infecções por arbovírus, Dengue, Ensaio de imunoadsorção enzimática, Testes sorológicos, Técnica indireta de fluorescência para anticorpo

## Abstract

**Objetivo:**

Avaliar o desempenho da imunofluorescência indireta no diagnóstico sorológico de dengue em uma população com alta prevalência de arboviroses.

**Métodos:**

Duzentas amostras de soro de pacientes com suspeita clínica de dengue foram testadas por ensaio imunoenzimático e imunofluorescência indireta mosaico BIOCHIP^®^. Foram calculados especificidade, sensibilidade e coeficiente Kappa. Nas amostras discordantes, realizou-se reação em cadeia da polimerase como método confirmatório.

**Resultados:**

Das 200 amostras, 20% foram positivas e 80% negativas para IgM antivírus da dengue no ensaio imunoenzimático. Das 40 positivas, 25% foram negativas na imunofluorescência indireta. Destas dez negativas, apenas 20% eram também negativas na reação em cadeia da polimerase. Das 160 negativas no ensaio imunoenzimático, 5% foram positivas na imunofluorescência indireta. Por fim, dentre as nove discordantes, 33% tiveram vírus da dengue detectado na reação em cadeia da polimerase. O coeficiente Kappa foi 0,70 (0,57-0,82). Sensibilidade e especificidade por imunofluorescência indireta foram, respectivamente, 75% e 94%. Para IgG antivírus da dengue, de 200 amostras, 15,5% foram positivas e 84,5% negativas no ensaio imunoenzimático. Das 31 positivas, 12,9% foram negativas na imunofluorescência indireta. Destas quatro discordantes, 25% apresentaram vírus da dengue não detectado na reação em cadeia da polimerase. Das 169 negativas, 8% foram positivas na imunofluorescência indireta. Destas, 64% foram positivas também na reação em cadeia da polimerase. O coeficiente Kappa foi 0,695 (0,56-0,83). Sensibilidade e a especificidade por imunofluorescência indireta foram, respectivamente, 87,1% e 91,7%.

**Conclusão:**

Ensaio imunoenzimático seria suficiente para diagnóstico sorológico de infecção aguda, não justificando a incorporação da imunofluorescência indireta. Substituir ensaio imunoenzimático pela imunofluorescência indireta poderia comprometer a sensibilidade para IgM. Contudo, a imunofluorescência indireta auxilia diferenciar três arboviroses simultaneamente, sendo vantajoso em epidemias concomitantes.

## INTRODUÇÃO

A dengue, uma arbovirose transmitida predominantemente por vetores da espécie *Aedes aegypti*, é um grave problema de saúde pública no Brasil, causando epidemias sazonais em praticamente todo o território nacional.^(^^1^^)^ Foram registrados, no país, 572.308 casos prováveis em 2014, 1.621.797 em 2015, 1.483.623 em 2016, 251.711 em 2017 e 265.934 em 2018.^(^^2^^–^^4^^)^ Todos os quatro sorotipos do vírus da dengue são encontrados no Brasil.

O diagnóstico laboratorial de dengue envolve principalmente técnicas como isolamento viral, identificação de anticorpos específicos para o vírus da dengue (DENV) pelos testes sorológicos, identificação direta do RNA viral e detecção do antígeno NS1.^(^^5^^–^^7^^)^

O diagnóstico sorológico da infecção aguda se baseia na detecção da imunoglobulina M (IgM) específica para DENV, que é detectável, em 93% dos casos, entre 6 a 10 dias após o aparecimento da febre.^(^^8^^)^ A imunoglobulina G (IgG) específica para DENV pode ser identificada em infecções atuais se, no momento da realização do teste, já tiver ocorrido a soroconversão, sendo útil para identificação de infecções passadas de dengue. O teste de avidez de IgG auxilia na diferenciação entre infecção primária ou secundária de dengue.^(^^6^^)^

O ensaio imunoenzimático (ELISA - *Enzyme-Linked Immunosorbent Assay*) se tornou o teste sorológico mais utilizado nos laboratórios clínicos. É um teste simples, rápido e que requer poucos equipamentos de alta tecnologia.^(^^9^^–^^11^^)^ Durante uma epidemia, o ELISA consegue determinar rapidamente o quão difundida a transmissão se tornou. Em áreas endêmicas de dengue, esse teste pode ser usado para rastreio de um grande número de amostras sorológicas a baixo custo.^(^^9^^)^

A imunofluorescência indireta é outro método sorológico que pode ser usado para identificar IgM e IgG específicos para dengue, e há poucos estudos^(^^12^^,^^13^^)^ que avaliam o uso desse teste. A maioria dos testes sorológicos disponíveis nos laboratórios clínicos brasileiros foi desenvolvida fora do país, com estudos de validação frequentemente realizados em populações de baixa prevalência da doença e que não experimentaram surtos concomitantes de outras arboviroses, como Zika ou Chikungunya, que podem levar a resultados falso-positivos de dengue por reação cruzada de anticorpos, dificultando seu diagnóstico.^(^^14^^)^

Durante o surto epidêmico de dengue em 2016, um novo teste comercial de imunofluorescência indireta (IFI) mosaico BIOCHIP^®^, que promete diferenciar sorologicamente infecções por dengue, Zika e Chikungunya, desenvolvido na Alemanha, foi lançado no mercado brasileiro, competindo com os testes de ELISA utilizados rotineiramente há mais tempo.

## OBJETIVO

Avaliar o desempenho diagnóstico de um teste de imunofluorescência indireta para o diagnóstico sorológico de dengue em uma população com alta prevalência de infecções por arbovírus comparando com o teste sorológico ELISA.

## MÉTODOS

Foram utilizadas 200 amostras de soro provenientes da rotina do Laboratório Clínico do Hospital Israelita Albert Einstein (HIAE), colhidas no ano de 2014 e encaminhadas ao laboratório, após suspeita clínica de infecção por dengue. As amostras foram caracterizadas à época como negativas ou positivas para dengue por ELISA (Foccus, EUA). Todas as amostras foram testadas pelo teste de IFI Mosaico BIOCHIP^®^ (Euroimmun, Alemanha).

Utilizando ferramentas estatísticas adequadas (software EP Evaluator), foram calculadas as probabilidades pré-teste: sensibilidade (capacidade que o teste diagnóstico apresenta de detectar os indivíduos verdadeiramente positivos) e especificidade (capacidade que o teste diagnóstico tem de detectar os verdadeiros negativos). Também foram calculadas probabilidades pós-teste: valor preditivo positivo (proporção de pacientes com teste positivo que efetivamente têm a doença de acordo com o teste padrão-ouro), valor preditivo negativo (proporção de pacientes que são negativos no teste e que efetivamente não têm a doença de acordo com o teste padrão-ouro). Por fim, foram calculados a acurácia, que é a probabilidade de o teste fornecer resultados corretos, e o coeficiente Kappa, uma medida de concordância entre dois métodos ajustada pela chance, ou seja, informa a proporção de concordância não aleatória, variando de −1 a 1, sendo, 0,00 para nenhuma concordância, 0,00-0,20 para concordância fraca, 0,21-0,40 para concordância sofrível, 0,41-0,60 para concordância regular, 0,61-0,80 para boa concordância, 0,81-0,99 para ótima concordância e 1 para concordância perfeita.^(^^15^^,^^16^^)^

Nas amostras discordantes entre os dois métodos, realizou-se a pesquisa do vírus por reação em cadeia da polimerase (PCR) como método diagnóstico confirmatório. O PCR é um teste molecular que faz uma detecção quantitativa do RNA do DENV, sendo considerado o padrão-ouro, uma vez que comprova a presença viral no organismo. Contudo, apresenta desvantagem, como possuir um alto custo, o que impede seu uso rotineiro como rastreio nos laboratórios.^(^^5^^)^

Este estudo foi aprovado pelo Comitê de Ética em Pesquisa do Hospital Israelita Albert Einstein, CAAE: 83521718.5.0000.0071, parecer número 2.909.625.

## RESULTADOS

Das 200 amostras estudadas, 40 (20%) foram classificadas como positivas e 160 (80%) como negativas para anticorpos IgM contra o DENV (anti-DENV), empregando-se o teste de referência ELISA.

Entre as 40 amostras positivas, 10 (25%) foram negativas no teste de IFI; destas 10 amostras discordantes, apenas 2 (20%) eram também negativas para dengue ao PCR. Por outro lado, das 160 amostras negativas no teste de referência ELISA, 9 (5%) foram positivas quando se empregou o teste de IFI; dessas 9 discordantes, 3 (33%) tiveram DENV detectado ao PCR (Tabela 1).

**Tabela 1:** Detecção de anticorpos IgM antivírus da dengue por meio do teste de ELISA e da imunofluorescência indireta

Imunofluorescência indireta IgM	ELISA IgM		Total
	Positivo	Negativo	
Positivo	30	9	39
Negativo	10	151	161
Total	40	160	200

Aplicando-se o teste Kappa ao conjunto de resultados dos dois ensaios, observou-se índice de concordância de 0,7 (0,572-0,829). A sensibilidade e a especificidade relativas da IFI foram, respectivamente, 75% e de 94%. O valor preditivo positivo da IFI foi 76,9%, o valor preditivo negativo da IFI foi 93,7% e a acurácia, 90,5%.

Para anticorpos IgG anti-DENV, de 200 amostras de soro, 31 (15,5%) foram classificadas como positivas e 169 (84,5%) como negativas, empregando-se o teste de ELISA. Das 31 amostras positivas, 4 (12,9%) foram negativas no teste de IFI; dessas 4 discordantes, apenas 1 (25%) apresentou DENV não detectado ao PCR. Por outro lado, das 169 amostras negativas, 14 (8%) foram positivas no teste de IFI avaliado; destas 14 discordantes, 9 (64%) foram positivas também ao PCR (Tabela 2).

**Tabela 2:** Detecção de anticorpos IgG antivírus da dengue. Comparação entre resultados obtidos empregando-se ELISA e imunofluorescência indireta

Imunofluorescência Indireta IgG	ELISA IgG		Total
	Reagente	Não reagente	
Reagente	27	14	41
Não reagente	4	155	159
Total	31	169	200

Aplicando-se o teste Kappa ao conjunto de resultados dos dois ensaios, observou-se índice de concordância de 0,695 (0,563-0,83). A sensibilidade e a especificidade relativas da IFI foram, respectivamente, de 87,1% e de 91,7%. O valor preditivo positivo da IFI foi 65,8%, o valor preditivo negativo da IFI foi 97,4% e a acurácia, 91%.

## DISCUSSÃO

A concordância, verificada pelo índice Kappa, entre o novo teste realizado por IFI e o teste utilizado como referência ELISA foi aceitável, demonstrando bom desempenho do novo teste.

Entretanto, a comparação com os resultados de PCR, método utilizado como confirmatório da presença de antígenos virais nas amostras com resultados discordantes entre os dois métodos, demonstrou maior concordância com o ELISA na maioria dos casos, exceto nos casos negativos por ELISA e positivos por IFI para anticorpos IgG anti-DENV (falso-positivos IgG), em que o resultado do PCR foi 64% coincidente com a imunofluorescência indireta.

## CONCLUSÃO

A imunofluorescência indireta possui desempenho aceitável, mas, para as situações de relevância clínica, no diagnóstico de infecção aguda (detecção de anticorpos IgM), a utilização apenas do teste de ELISA seria suficiente para o diagnóstico sorológico, não se justificando a incorporação de mais um método. Por outro lado, a substituição do teste de ELISA pela imunofluorescência indireta poderia comprometer a sensibilidade diagnóstica, aumentando o número de amostras falso-negativas para IgM.
